# Comprehensive insights into pathogen distribution, clinical features, and outcomes in pediatric severe pneumonia

**DOI:** 10.3389/fcimb.2025.1681950

**Published:** 2026-01-07

**Authors:** Chunyun Fu, Xiangjun Lu, Jiangyang Zhao, Ya Huang, Qiang Huang, Lishai Mo, Yanhua Feng, Wenting Tang, Cuihong Lu, Mengjun Li, Yubing Wei, Ruting Chen, Guangbing Liu, Jialing Ruan, Huiping Huang, Qifei Li, Jie Tan

**Affiliations:** 1 Medical Science Laboratory, Children’s Hospital, Maternal and Child Health Hospital of Guangxi Zhuang Autonomous Region, Nanning, China; 2 Department of Pediatric Respiratory Medicine, Children’s Hospital, Maternal and Child Health Hospital of Guangxi Zhuang Autonomous Region, Nanning, China; 3 Division of Neonatology, Department of Pediatrics, University of Miami Miller School of Medicine and Holtz Children’s Hospital, Jackson Health System, Miami, FL, United States

**Keywords:** children, infection, pathogens, severe pneumonia, TNGS

## Abstract

**Objective:**

Pediatric severe pneumonia is a life-threatening condition with high incidence and mortality rates. This study provides a comprehensive analysis of pathogen distribution, infection patterns, clinical manifestations, and prognosis associated with the disease.

**Methods:**

This study involved 227 children with severe pneumonia and a control group of 227 with non-severe pneumonia. Targeted next-generation sequencing (tNGS) was applied to identify respiratory pathogens.

**Results:**

The median age of children with severe pneumonia was 12 months, markedly younger than the median of 24 months in the non-severe group. The tNGS identified suspected pathogenic microorganisms in 99.12% of the severe pneumonia cases, with predominant pathogens including Human Respiratory Syncytial Virus (33.92%), Cytomegalovirus (33.04%), *Mycoplasma pneumoniae* (24.67%), and Rhinovirus (23.79%). Clinically, those with severe pneumonia exhibited markedly elevated procalcitonin levels and an increased prevalence of fever, wheezing, and dyspnea, alongside longer hospital stays, more protracted fever, and significantly higher hospitalization costs compared to their non-severe counterparts. Complications, including respiratory and other systemic issues, occurred in 96.04% and 59.47% of the severe pneumonia group, respectively, both significantly higher than in the non-severe pneumonia group; 99.12% required respiratory support and significantly more (56.83%) were admitted to the ICU, with a 9.69% incidence of poor outcomes.

**Conclusion:**

Children with severe pneumonia tend to be younger than those with non-severe pneumonia. While significant differences are observed in the distribution of pathogens, complications, clinical manifestations, and prognosis between the two groups, no significant differences are noted in the number of infecting microorganisms or infection patterns.

## Introduction

Pneumonia is a major cause of death in children under five, resulting in about one million deaths worldwide each year (Yamaguchi et al., 2021; Abeja et al., 2022). Clinically, pediatric pneumonia typically presents with fever, cough, tachypnea, and auscultatory findings such as crackles or diminished breath sounds, which may be accompanied by chest retractions or hypoxemia in severe cases. From a diagnostic perspective, the confirmation of pneumonia relies on a combination of clinical features and imaging evidence, with chest X-ray or computed tomography demonstrating pulmonary infiltrates consistent with infection, supported by laboratory findings such as leukocytosis and positive pathogen detection (Bradley et al., 2011). Severe pneumonia is a critical illness in pediatrics, accounting for 7% to 16% of pediatric pneumonia cases (Brooks et al., 2021; Chen et al., 2021). Despite general improvements in living conditions, improved nutrition, and better vaccines, the incidence and mortality rates of severe pneumonia remain high, posing a serious threat to child health, particularly in low-and-middle-income countries (Wang et al., 2020; Atwell et al., 2022).

Previous studies have established that co-infections are common in severe pediatric pneumonia, and that early pathogen identification coupled with effective treatment is essential for achieving favorable patient outcomes (Qu et al., 2022; Liu et al., 2023; Tan et al., 2025). However, few studies in the past decade have comprehensively profiled the full spectrum of causative pathogens—including viruses, bacteria, and fungi—or systematically analyzed their co-infection patterns in this population (Liu et al., 2023; Zhang et al., 2024). To address this gap, we employed tNGS to detect pathogens in 227 children with severe pneumonia and 227 with non-severe pneumonia. This study provides a comprehensive comparison of the two cohorts regarding pathogen distribution, infection patterns, laboratory findings, clinical manifestations, imaging features, complications, and prognosis. These findings offer valuable insights to guide clinical diagnosis, therapeutic strategy, and management of pediatric severe pneumonia.

## Materials and methods

### Study population

This study enrolled 227 children with severe pneumonia and 227 randomly sampled children with non-severe pneumonia as controls from the Maternal and Child Health Hospital of Guangxi Zhuang Autonomous Region between July and November 2023.

### Definition of severe and non-severe pneumonia

Severe pneumonia was defined as radiologically confirmed pneumonia accompanied by at least one major criterion, including: general danger signs such as impaired consciousness, convulsions, inability to feed, dehydration, or severe vomiting; respiratory distress and hypoxemia manifested by central cyanosis, SpO_2_ ≤ 92% in room air, grunting, severe tachypnea, or chest wall indrawing; respiratory failure requiring non-invasive or invasive mechanical ventilation; radiological evidence of complications including multi-lobar involvement, pleural effusion, or necrotizing pneumonia; or systemic complications such as hemodynamic instability, shock, heart failure, acute kidney injury, or other organ dysfunction.

For this study, organ dysfunction was specifically defined as meeting one or more of the following criteria: cardiovascular dysfunction requiring vasoactive agents or presenting with septic shock; neurological dysfunction indicated by a Glasgow Coma Score ≤11 or its rapid decline of ≥3 points; renal dysfunction characterized by serum creatinine ≥2 times the age-specific upper limit of normal or requiring renal replacement therapy; hepatic dysfunction manifested as hyperbilirubinemia, coagulopathy, or transaminase elevation >5 times the upper limit of normal; or hematological dysfunction evidenced by disseminated intravascular coagulation or severe thrombocytopenia. Notably, isolated mild transaminase elevations (≤5 times the upper limit of normal) without other hepatic impairment were excluded from this classification.

Non-Severe Pneumonia was diagnosed in children with clinical symptoms and radiological findings consistent with pneumonia who did not meet any of the criteria for severe pneumonia. These patients typically exhibited fever, cough, and tachypnea without signs of severe respiratory distress, hypoxemia, or major systemic complications.

### Pathogen-targeted next-generation sequencing

The respiratory tract specimens were collected according to the standard clinical procedure and guideline (Pediatric Bronchoscopy Collaborative Group TSGoRDTSoPCMA, 2009; Zhan et al., 2022). The collection consisted of 276 bronchoalveolar lavage fluid (BALF) samples (137 from severe and 139 from non-severe cases), 146 throat swabs (75 severe, 71 non-severe), and 32 sputum samples (15 severe, 17 non-severe). Nucleic acid from the samples was extracted and purified using MagPure Pathogen DNA/RNA Kit (R6672-01B, Magen, Guangzhou, China) according to the manufacturer’s instructions. A multiplex PCR library system (Respiration100TM, KingCreate, Guangzhou, China) and the next-generation sequencing technology were applied to detect 198 respiratory pathogens (80 bacteria, 79 viruses, 32 Fungi, 7 Other) (**Supplementary Table 1**). The methods and workflows of tNGS have been described in detail previously (Fu et al., 2025). Subsequently, the clinical data of the patients were comprehensively evaluated independently by two experienced clinicians to determine the relevance of pulmonary infections and clinical potential pathogens. The evaluation included the patient’s medical history, clinical symptoms, routine microbiological tests, inflammatory responses, epidemiology, imaging findings, tNGS results, and laboratory test results. When two doctors have a different opinion, further consult the senior doctor to reach a consensus.

### Differentiating pathogenic infection from colonization

To accurately distinguish true infection from colonization, we established a comprehensive diagnostic framework integrating four critical domains: (1) Host Susceptibility, including immunocompromised status (e.g., neutropenia, HIV, solid organ transplantation) or prematurity; (2) Microbiological Evidence, encompassing high microbial load by tNGS, low Ct values in PCR, microscopic identification of invasive forms, and recovery of pathogens from sterile sites; (3) Clinical-Radiological-Inflammatory Correlation, evidenced by new/worsening pulmonary infiltrates, ground-glass opacities, viral interstitial patterns, or elevated biomarkers (e.g., PCT, CRP, GM); and (4) Therapeutic Response, defined as clinical improvement or virological clearance following targeted antimicrobial therapy. This multi-parameter system enhances the precision of clinical attribution for detected microorganisms.

### Statistical analysis

All statistical analyses were performed using IBM SPSS Statistics (version 26.0). Continuous variables with a normal distribution are presented as the mean ± standard deviation (SD) and were compared using the independent-samples t-test. Continuous variables with a skewed distribution are presented as the median (interquartile range, IQR) and were compared using the Mann-Whitney U test. Categorical variables are expressed as frequencies and percentages (n, %) and were compared using the Chi-square test. A two-tailed P value of < 0.05 was considered statistically significant.

## Results

### Basic information of the children

A total of 227 children hospitalized with severe pneumonia were collected, including 146 males and 81 females, with a male-to-female ratio of 1.80:1. The age distribution ranged from 1 month to 12 years and 1 month, with a median age of 12 months. Among these children, 97 were under 12 months, 56 were aged 12 to 24 months, 21 were aged 24 to 36 months, and 53 were over 36 months. In contrast, among the 227 children with non-severe pneumonia, there were 148 males and 79 females, resulting in a male-to-female ratio of 1.87:1. The median age of children with non-severe pneumonia was 24 months, significantly older than that of children with severe pneumonia (*P* < 0.001) (**Table 1**).

**Table 1:** Quantitative clinical and laboratory characteristics by pneumonia severity.

Clinical features	Non-severe pneumonia	Severe pneumonia	*P* value
Quantitative data [M(IQR)]	N=227	N=227	
Age(months)	24.00(40.00)	12.00(28.00)	***<0.001
WBC(×10^9^/L)	9.10(4.95)	10.7(7.37)	**0.004
NEU(%)	45.50(27.93)	56.85(31.07)	***<0.001
LYM(%)	44.10(25.48)	32.95(26.95)	***<0.001
RBC(×10^12^/L)	4.50(0.5)	4.3(0.8)	***<0.001
Eos(%)	1.00(2.20)	0.50(1.60)	***<0.001
PLT(×10^9^/L)	353.00(153.00)	401.50(215.50)	**0.002
CRP (mg/L)	3.16(11.60)	5.29(9.21)	0.180
PCT(ng/ml)	0.080(0.16)	0.13(0.25)	***<0.001
SAA(mg/L)	17.18(81.10)	32.13(77.14)	0.535
D-dimer(ng/ml FEU)	367.50(397.50)	490.00(640.00)	***<0.001
LDH(U/L)	314.00(93.00)	391.00(105.00)	0.127
CK(U/L)	90.50(63.75)	77.00(86.00)	0.128
CK-MB(U/L)	25.50(13.00)	25.00(16.00)	0.819
AST(U/L)	34.00(16.00)	33.00(16.00)	0.982
ALT(U/L)	16.00(10.75)	17.00(13.00)	0.114
URE(umol/L)	25.88(12.88)	22.00(10.00)	***<0.001
CYs-C(mg/L)	0.88(0.28)	0.93(0.31)	0.139
Length of hospitalization (days)	7.00(3.5)	14.00(12.00)	***<0.001
Treatment expense(CNY)	8919.61(4717.38)	36844.11(31024.53)	***<0.001
Duration of fever (days)	3.00(6.00)	8.00(9.25)	***<0.001

*P < 0.05, **P < 0.01 and ***P < 0.001 represent statistically significant differences. WBC, White blood cell; NEU, Neutrophil; LYM, Lymphocyte; Mon, Monocyte; Eos, Eosinophil; Baso, Basophil; RBC, Red Blood Cell; PLT, Platelet; CRP, C-reactive protein; PCT, Procalcitonin; SAA, Serum Amyloid A; ESR, Erythrocyte Sedimentation Rate; LDH, Lactic acid dehydrogenase; CK, Creatine kinase; CK-MB, Creatine kinase-MB; AST, Aspartate aminotransferase; ALT, Alanine Aminotransferase; URE, Urea; CYs-C, Cystatin C; CNY, Chinese Yuan. The bold values indicate statistical significance (P < 0.05).

### Number of microorganisms detected

**Figure 1:**
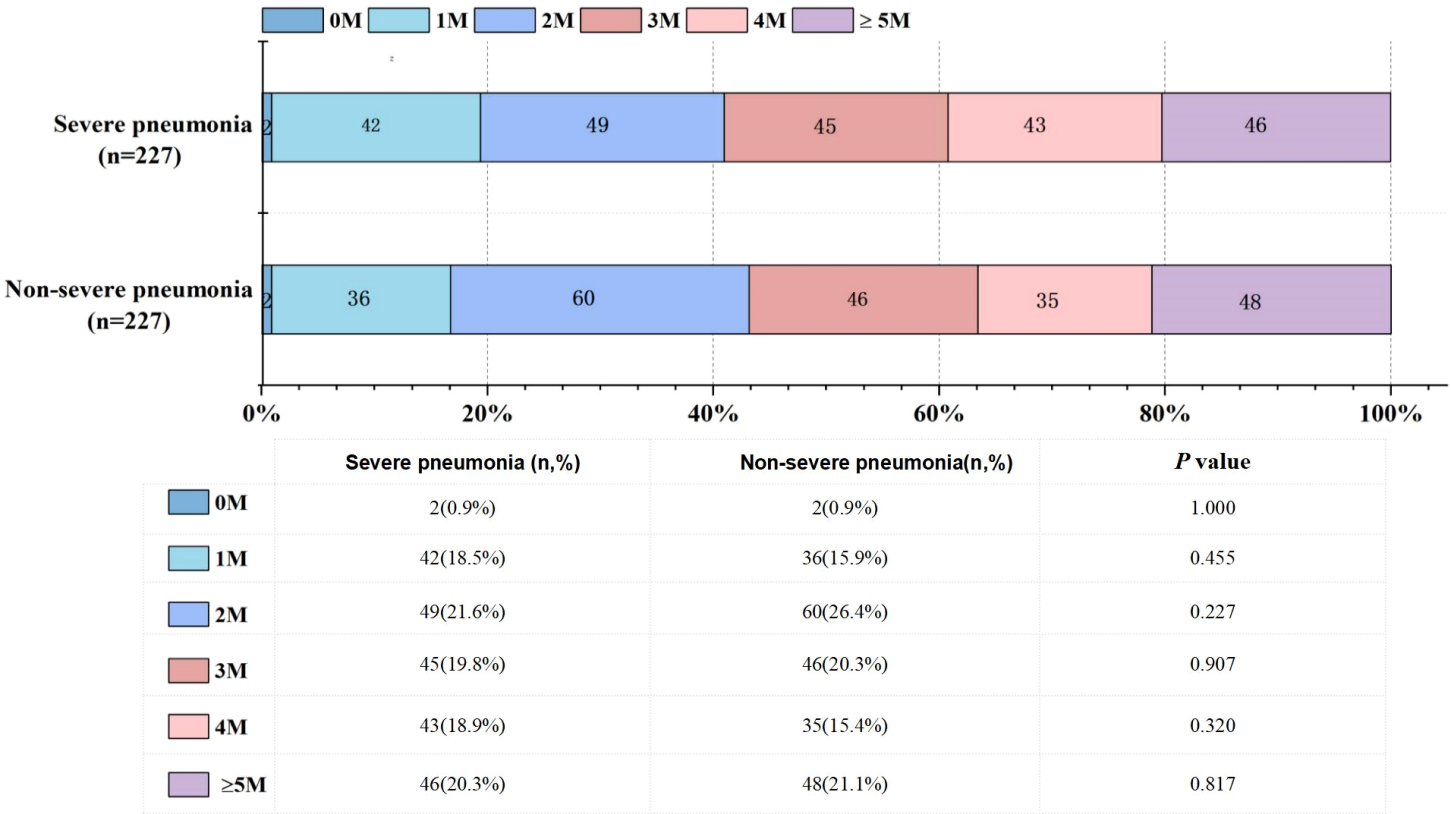
Potential pathogen counts in severe vs. non-severe pediatric pneumonia. 0M: no microorganism detected; 1M: one microorganism detected; 2M: two microorganisms detected; 3M: three microorganisms detected; 4M: four microorganisms detected; ≥5M: five or more microorganisms detected.

Of the 227 children with severe pneumonia, tNGS identified suspected pathogenic microorganisms in 225 cases, yielding a positive detection rate of 99.12% with only 2 cases testing negative. Specifically, there were 42 cases of infections with a single microorganism, 49 cases of co-infections with 2 microorganisms, 45 cases of co-infections with 3 microorganisms, 43 cases of co-infections with 4 microorganisms, and 46 cases of co-infections with 5 or more microorganisms (**Figure 1**). Among the 227 children with non-severe pneumonia, tNGS also detected suspected pathogenic microorganisms in 225 cases (99.12%), with only 2 cases testing negative. Specifically, 36 cases had infections with a single microorganism, 60 cases had co-infections with 2 microorganisms, 46 cases had co-infections with 3 microorganisms; 35 cases had co-infections with 4 microorganisms, and 50 cases had co-infections with 5 or more microorganisms (**Figure 1**). There were no significant differences in the proportion of various numbers of microorganisms detected between children with severe pneumonia and those with non-severe pneumonia (**Figure 1**).

### Microorganism distribution

**Figure 2:**
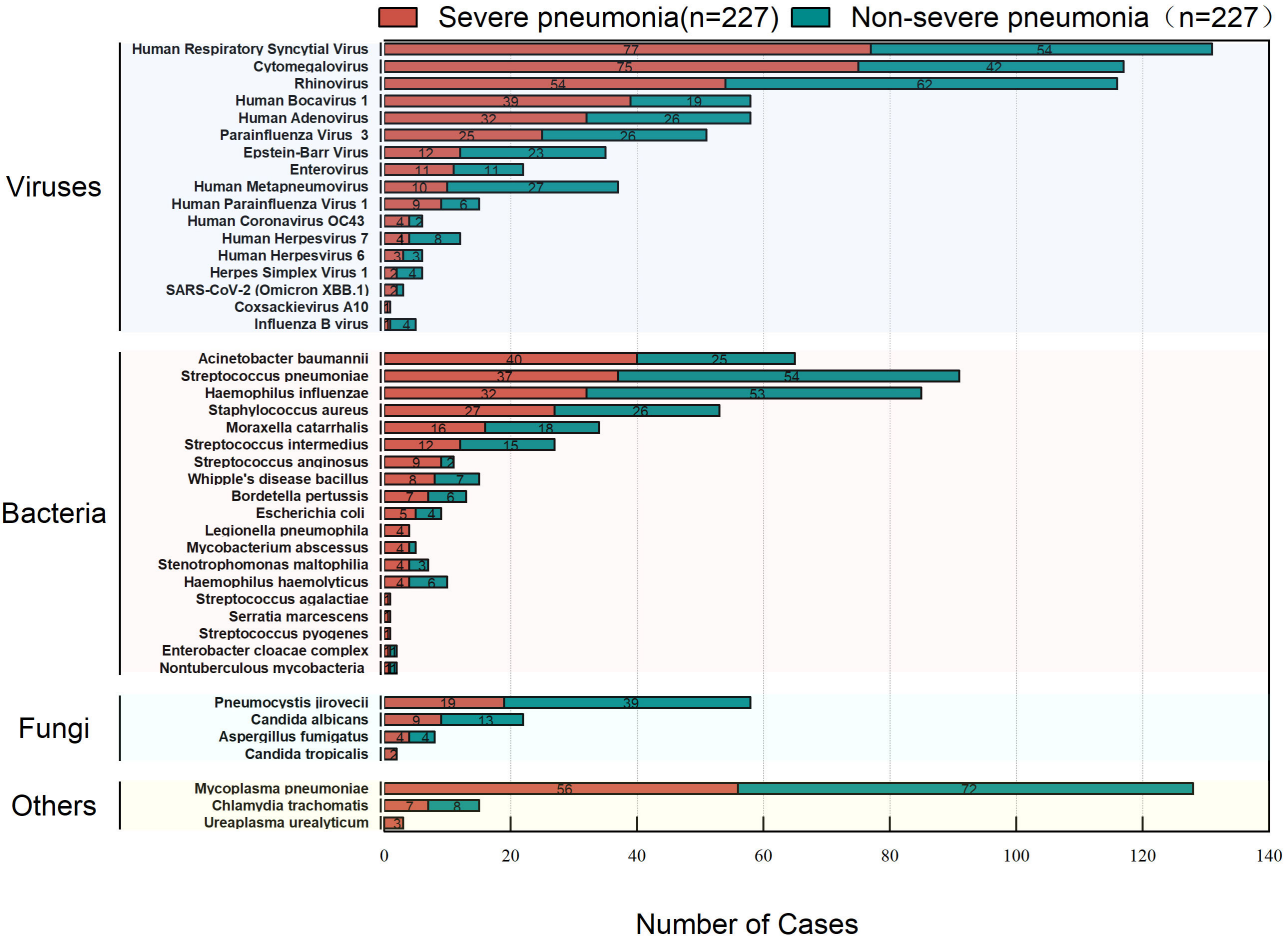
Microbial spectrum in severe vs. non-severe pediatric pneumonia.

A total of 43 microorganisms were detected in the 227 children with severe pneumonia. The top six microorganisms, listed in descending order of detection rate, were Human Respiratory Syncytial Virus (33.92%, 77/227), Cytomegalovirus (33.04%, 75/227), *Mycoplasma pneumoniae* (24.67%, 56/227), Rhinovirus (23.79%, 54/227), *Acinetobacter baumannii* (17.62%, 40/227), and Human Bocavirus type 1 (17.18%, 39/227). The ranking for the top six most prevalent microorganisms in children with non-severe pneumonia differed from that in the severe pneumonia group, with the rank being *Mycoplasma pneumoniae* (31.72%, 72/227), Rhinovirus (27.31%, 62/227), *Streptococcus pneumoniae* (23.79%, 54/227), Human Respiratory Syncytial Virus (23.79%, 54/227), *Haemophilus influenzae* (23.35%, 53/227), and Cytomegalovirus (18.50%, 42/227) (**Figure 2**).

**Figure 3:**
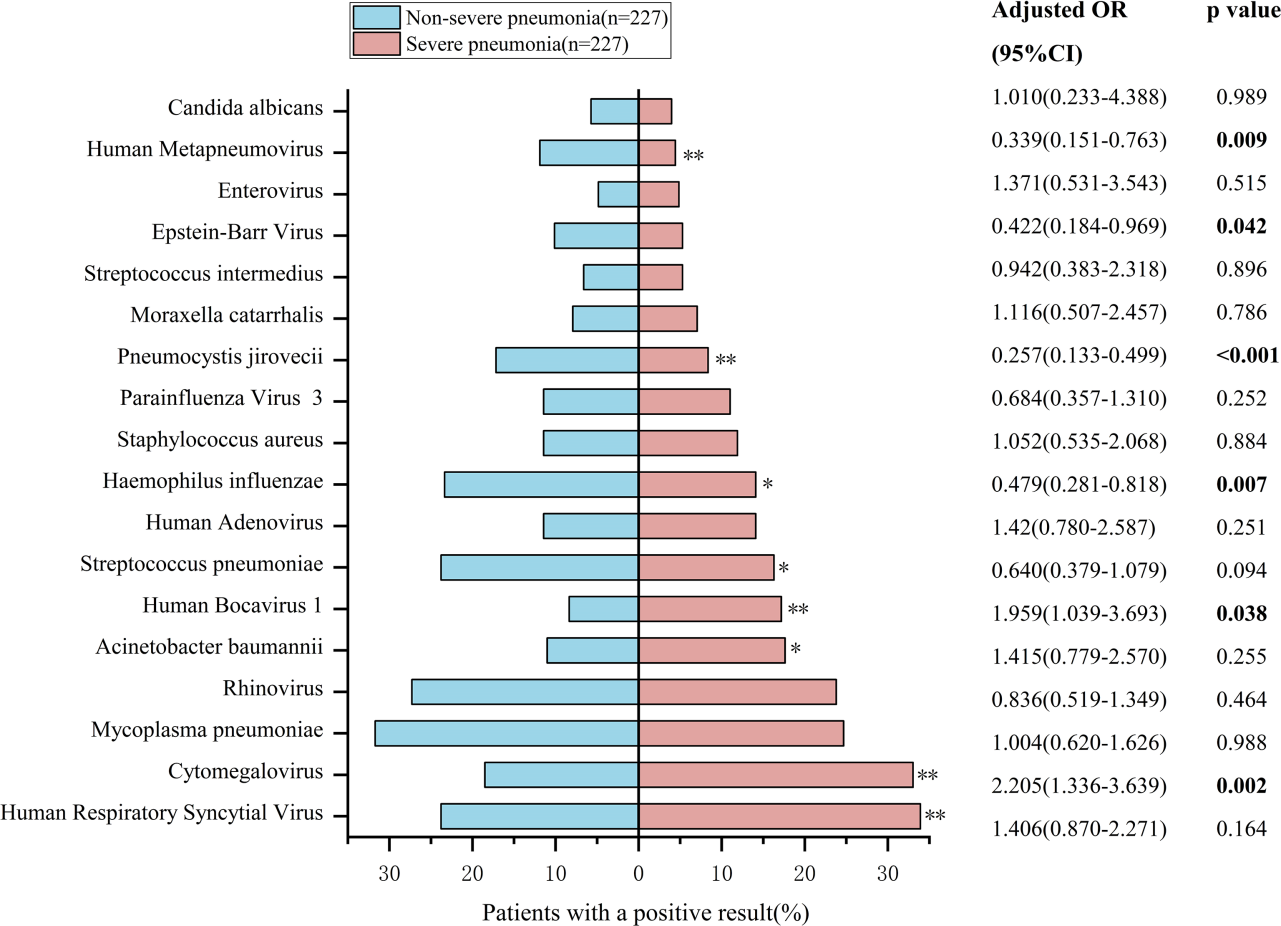
Top pathogens in severe vs. non-severe pediatric pneumonia. Comparison of the detection rates for the top 18 suspected pathogenic microorganisms between severe pneumonia and non-severe pneumonia. Bars represent positive rates. P values were determined by Chi-square test (*P < 0.05, **P < 0.01, ***P < 0.001). Odds ratios (ORs) with 95% confidence intervals (CIs) were determined by multivariable regression analysis, adjusted for gender, age, and the other 17 pathogens.

Among the top 18 microorganisms detected in children with severe pneumonia, chi-square test indicated significant differences in the detection rates of human respiratory syncytial virus, cytomegalovirus, *acinetobacter baumannii*, human bocavirus type 1, *Streptococcus pneumoniae*, *Haemophilus influenzae*, *Pneumocystis jirovecii*, and human metapneumovirus in children with severe pneumonia compared to those with non-severe pneumonia (*P* < 0.05). After adjusting for confounding factors (gender, age, and the other 17 pathogens), only cytomegalovirus, human bocavirus type 1, *Haemophilus influenzae*, *Pneumocystis jirovecii*, Epstein-Barr virus, and human metapneumovirus exhibited significantly different detection rates between the severe pneumonia and non-severe pneumonia groups (*P* < 0.05). Cytomegalovirus and human bocavirus type 1 were detected at higher rates in children with severe pneumonia than in those with non-severe pneumonia (**Figure 3**).

### Infection patterns

In children with severe pneumonia, the predominant infection patterns are mixed infections of viruses and bacteria (35.2%, 80/227) and single viral infections (11.9%, 27/227). Among non-severe pneumonia cases, mixed viral-bacterial infections accounted for 30.4% (69/227), followed by viral-bacterial-other pathogen co-infections at 10.1% (23/227). No significant differences were found in infection patterns between the severe and non-severe pneumonia groups (**Table 2**).

**Table 2:** Infection patterns in severe vs. non-severe pediatric pneumonia.

Types of infections	Severe pneumonia(N=227)	Non-severe pneumonia(N=227)	*P* value
Viral-Bacterial co-infection	80(35.2%)	69(30.4%)	0.272
Single viral infection	27(11.9%)	16(7.0%)	0.078
Viral-Bacterial-Others co-infection	23(10.1%)	23(10.1%)	1.000
Viral-Viral co-infection	21(9.3%)	16(7.0%)	0.391
Viral-Others co-infection	18(7.9%)	21(9.3%)	0.615
Viral-Bacterial-Fungal co-infection	11(4.8%)	15(6.6%)	0.419
Other infection	10(4.4%)	15(6.6%)	0.304
Viral-Fungal co-infection	9(4.0%)	16(7.0%)	0.150
Viral-Fungal-Others co-infection	6(2.6%)	2(0.9%)	0.285
Bacterial-Bacterial co-infection	6(2.6%)	3(1.3%)	0.501
Single bacterial infection	5(2.2%)	5(2.2%)	1.000
Viral-Bacterial-Fungal-Others co-infection	4(1.8%)	10(4.4%)	0.103
Bacterial-Others co-infection	3(1.3%)	9(4.0%)	0.079
Bacterial-Fungal co-infection	1(0.4%)	3(1.3%)	0.616
Fungal-Others co-infection	1(0.4%)	1(0.4%)	1.000
Bacterial-Fungal-Others co-infection	0(0%)	1(0.4%)	–

Other: *Mycoplasma pneumoniae* or *Chlamydia trachomatis*, *P* < 0.05 represent statistically significant differences. The bold values indicate statistical significance (P < 0.05).

### Clinical characteristics and laboratory tests

According to **Table 3**, children with severe pneumonia exhibited a significantly higher proportion of symptoms—fever, wheezing, and shortness of breath—compared to children with non-severe pneumonia. Compared to the non-severe pneumonia group, the severe pneumonia group had longer hospital stays, extended fever durations, and significantly higher treatment costs (**Table 1**). Laboratory tests indicated that the severe pneumonia group had significantly higher white blood cell (WBC) counts, neutrophil percentages, platelet (PLT) counts, D-dimer levels, and procalcitonin (PCT) levels compared to the non-severe pneumonia group. In contrast, the severe pneumonia group showed notably lower red blood cell (RBC) counts and lymphocyte percentages. Despite these variations, all median values for these tests remained within the normal reference ranges, except for the PCT levels, which exceeded the normal reference levels (**Table 1**).

**Table 3:** Categorical clinical and radiological characteristics by pneumonia severity.

Clinical features	Non-severe pneumonia	Severe pneumonia	*P* value
Categorical data, n(%)	N=227	N=227	
Gender (male)	148(65.2%)	146(64.3%)	0.884
Fever	162(71.4%)	209(92.1%)	***<0.001
Cough	213(93.8%)	208(91.6%)	0.336
Expectoration	185(81.5%)	181(79.7%)	0.635
Wheezing	76(33.5%)	162(71.4%)	***<0.001
Shortness of breath	37(16.3%)	184(81.1%)	***<0.001
Chest pain	0(0.0%)	2(0.9%)	0.479
Ear discharge	2(0.9%)	0(0.0%)	0.479
Running nose	85(37.4%)	82(36.1%)	0.770
Stomachache	2(0.9%)	5(2.2%)	0.446
Respiratory support	122(53.7%)	225(99.1%)	***<0.001
Computed tomography imaging			
Thickening of bronchial walls	168(74.0%)	148(65.2%)	0.041
Bilateral lobar consolidation	45(19.8%)	118(52.0%)	***<0.001
Atelectasis	6(2.6%)	30(13.2%)	**0.001
Pleural effusion	11(4.8%)	26(11.5%)	*0.011
Unilateral lobar consolidation	35(15.4%)	26(11.5%)	0.216
Pulmonary ground-glass opacity	3(1.3%)	8(3.5%)	0.127
Enlarged mediastinal lymph nodes	4(1.8%)	1(0.4%)	0.177
Bronchiectasis	3(1.3%)	0(0%)	0.082

*P < 0.05, **P < 0.01 and ***P < 0.001 represent statistically significant differences. The bold values indicate statistical significance (P < 0.05).

### Evaluation of the concordance among the three methods (tNGS, traditional PCR, and immunological detection methods)

To evaluate the concordance among tNGS, polymerase chain reaction (PCR), and IgM serology, we selected two common respiratory pathogens: Respiratory Syncytial Virus (RSV) and *Mycoplasma pneumoniae* (MP).

**Figure 4:**
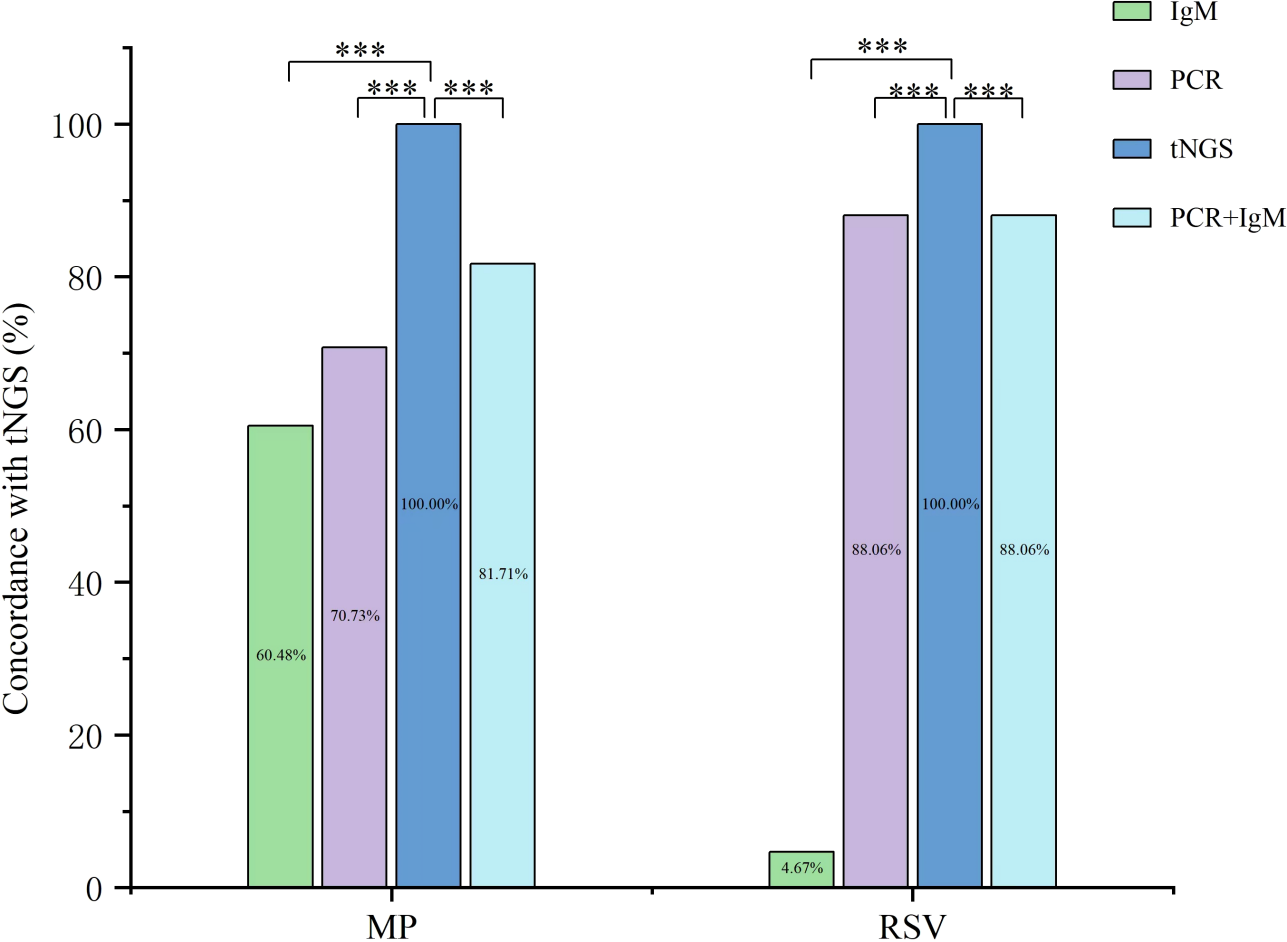
Concordance among detection methods for MP and RSV. Evaluation of the detection agreement between tNGS, traditional PCR, and IgM serology for *Mycoplasma pneumoniae* (MP) and respiratory syncytial virus (RSV). P values were derived from Chi-square test (***P < 0.001).

For the 127 MP cases identified by tNGS, the concordance rates of traditional methods were as follows: IgM serology was positive in 75 of 124 tested cases (60.48%), PCR was positive in 58 of 82 cases (70.73%), and the combination of both methods identified 67 of 82 cases (81.71%) (**Figure 4**).

Similarly, for the 126 RSV cases detected by tNGS, the concordance rates were: IgM serology in 5 of 107 cases (4.67%), PCR in 59 of 67 cases (88.06%), and the combination of both methods in 59 of 67 cases (88.06%) (**Figure 4**).

### Computed tomography imaging examination

Computed tomography imaging of 227 hospitalized children with severe pneumonia showed the following results: 148 cases (65.2%) had thickened bronchial walls, 118 cases (52.0%) exhibited bilateral lobar consolidation, 30 cases (13.2%) presented with atelectasis, and there were 26 cases (11.5%) each of pleural effusion and unilateral lobar consolidation. Additionally, eight cases (3.5%) showed pulmonary ground-glass opacity, while one case had enlarged hilum and mediastinal lymph nodes (**Table 3**). There is a significant difference in the occurrence of bilateral lobar consolidation, atelectasis, and pleural effusion, with a higher proportion found in patients with severe pneumonia compared to those with non-severe pneumonia (**Table 3**).

### Respiratory support

**Figure 5:**
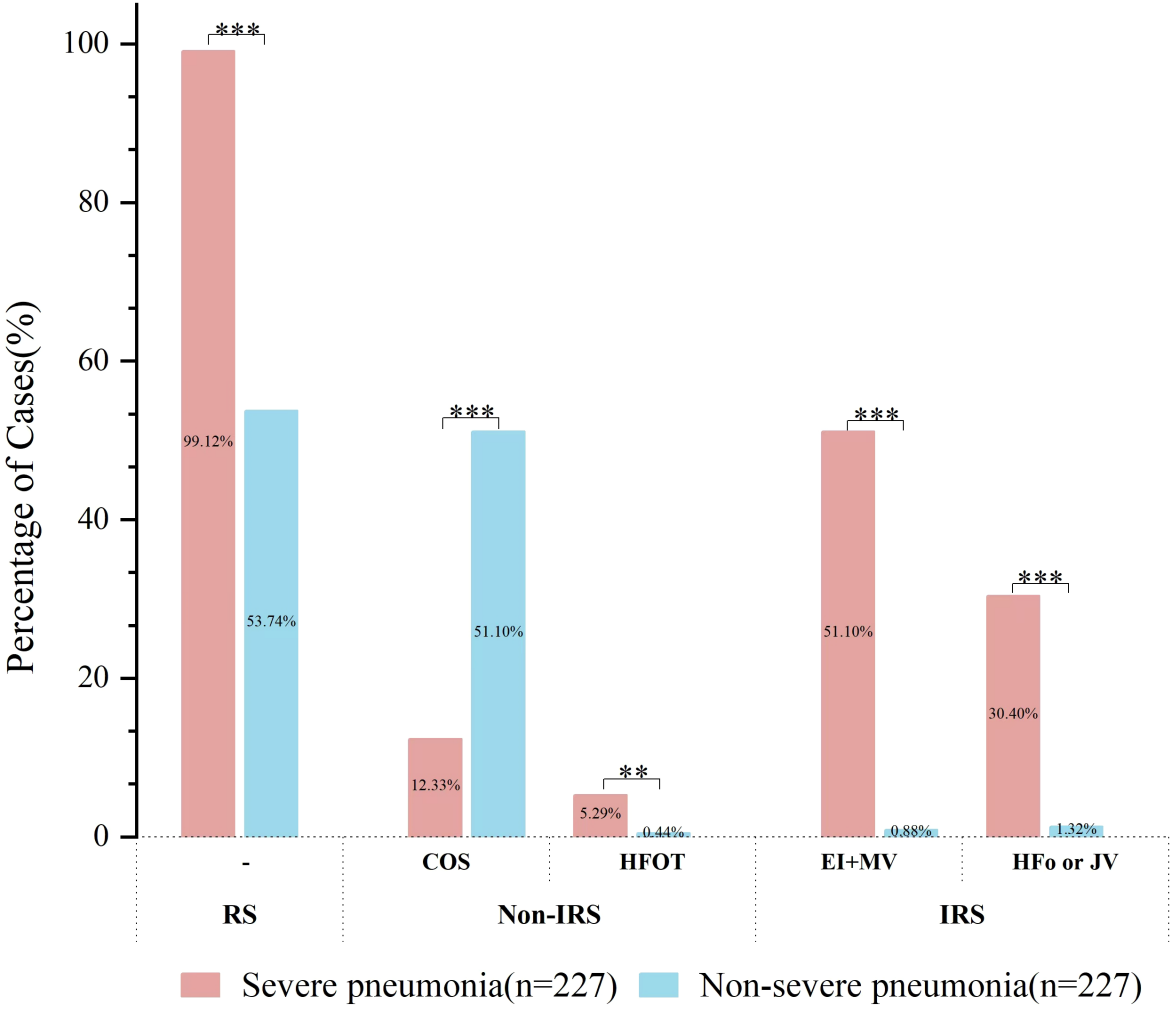
Respiratory support in severe vs. non-severe pediatric pneumonia. Comparison of respiratory support between the two groups. RS, respiratory support; IRS, invasive respiratory support; COS, Central oxygen supply; HFOT, High-frequency oxygen therapy; EI, Endotracheal intubation; MV, Mechanical ventilation; HFo, High-frequency oscillatory; JV, Jet ventilation. P values were determined by Chi-square test (**P < 0.01, ***P < 0.001).

Among 227 hospitalized children with severe pneumonia, 225 (99.12%) needed respiratory support. This support comprised 40 cases (17.62%) of non-invasive respiratory aid, which included 28 children using central oxygen supply and 12 receiving high-frequency oxygenation. Additionally, 185 cases (81.50%) required invasive respiratory support. This included 116 children who underwent endotracheal intubation or tracheostomy with mechanical ventilation, and 69 children who received high-frequency oscillatory or jet ventilation (**Figure 5**).

Among the 227 hospitalized children diagnosed with non-severe pneumonia, 122 (53.74%) required respiratory support. This consisted of 117 cases (51.54%) that received non-invasive support, including 116 with central oxygen supply and 1 with high-frequency oxygenation. Additionally, 5 cases (2.20%) required invasive respiratory support (these 5 patients had severe underlying comorbidities, and ventilatory support was a standard measure for acute exacerbation of these conditions), which included 2 cases with endotracheal intubation and mechanical ventilation, and 3 cases with high-frequency oscillatory or jet ventilation (**Figure 5**).

The proportion of children with severe pneumonia requiring invasive respiratory support was significantly higher (81.50%, 185 cases) compared to the non-severe pneumonia group (2.20%, 5 cases) (**Figure 5**).

### Complications

**Figure 6:**
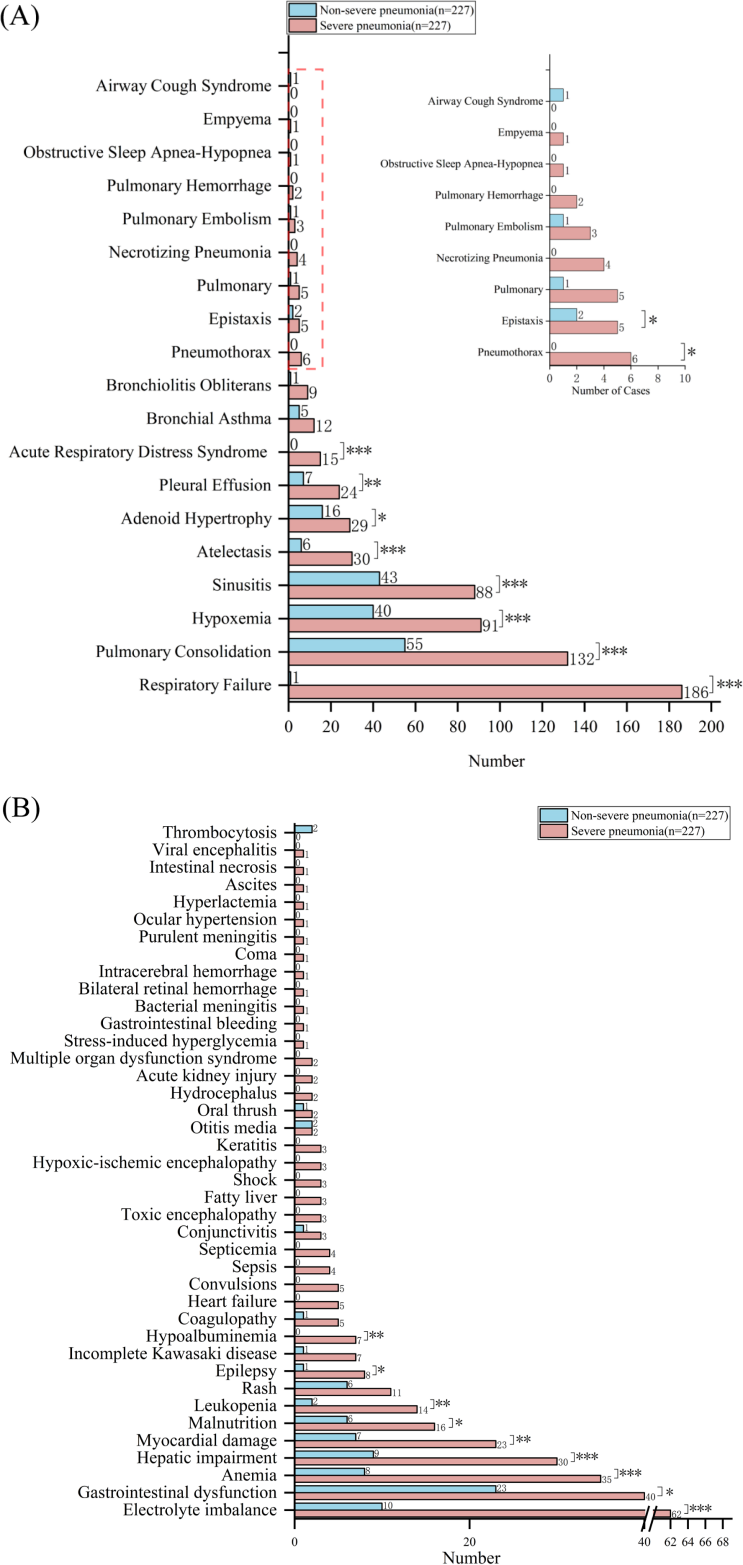
Complication profiles in severe vs. non-severe pediatric pneumonia. **(A)** Respiratory and **(B)** non-respiratory complications in children with severe versus non-severe pneumonia. Bars represent case numbers for each complication. P values were determined by Chi-square test (*P < 0.05, **P < 0.01, ***P < 0.001).

Among the 227 hospitalized children with severe pneumonia, 218 cases (96.04%) experienced respiratory complications. The most common complication was respiratory failure (186 cases), followed by pulmonary consolidation (132), hypoxemia (91), and sinusitis (88) (**Figure 6A**). Among the 227 hospitalized children with non-severe pneumonia, 127 cases (55.95%) had respiratory complications, which was significantly lower than the incidence in the severe pneumonia group, with the top 4 being pulmonary consolidation (55 cases), sinusitis (43), hypoxemia (40), and adenoid hypertrophy (16) (**Figure 6A**).

Among 227 hospitalized children with severe pneumonia, 135 cases (59.47%) experienced complications in other systems. The five most common complications were electrolyte disorders (62 cases), gastrointestinal dysfunction (40), anemia (35), liver damage (30), and myocardial damage (23) (**Figure 6B**). In contrast, among 227 hospitalized children with non-severe pneumonia, only 67 cases (29.52%) had complications in other systems, indicating a significantly lower incidence than that observed in the severe pneumonia group. The five most common complications in the non-severe pneumonia group were gastrointestinal dysfunction (23 cases), electrolyte disorders (10), liver damage (9), anemia (8), and myocardial damage (7) (**Figure 6B**).

### Prognosis

Among 227 hospitalized children with severe pneumonia, the median hospital stay was 14 days, significantly longer than the 7-day median stay for those with non-severe pneumonia. Of the 227 children, 129 (56.83%) required admission to the ICU for intensive care, a rate significantly higher than the zero cases in the non-severe pneumonia group.

After treatment, 205 (90.31%) of the 227 hospitalized children with severe pneumonia (90.31%) improved and were discharged, while 22 (9.69%) experienced poor outcomes. Among these cases, 19 children were transferred to another hospital at their families’ request due to unsatisfactory outcomes, while 3 children died due to ineffective treatment. Among the 227 hospitalized children with non-severe pneumonia, 223 (98.24%) improved and were discharged, only 4 (1.76%) experienced poor treatment responses, leading their families to request discharge. The rate of poor outcomes was significantly lower in the group with non-severe pneumonia compared to the group with severe pneumonia (*P* < 0.05).

## Discussion

Previous studies indicate that younger children, particularly those under one year of age, are more susceptible to severe pneumonia and tend to experience more severe clinical conditions during hospitalization (Raba et al., 2020; Srivastava et al., 2024). A retrospective study revealed that boys are significantly more prone to severe pneumonia than girls, with the highest incidence observed in children under two years of age (Goyal et al., 2021). Here, this study included 227 pediatric cases of severe pneumonia, comprising 97 children under 12 months and 56 children aged 12 to 24 months, totaling 153 children under two years of age, accounting for 67.40% of the cohort. The median age of these children was significantly younger than that of the non-severe pneumonia group, highlighting the need for special attention and monitoring of respiratory infections in this population, likely due to their underdeveloped immune systems. Additionally, males were more frequently affected than females, consistent with findings from previous research (Sultana et al., 2024; Zhang et al., 2024).

In children with severe pneumonia, single microorganism infections were identified in only 42 cases (18.50%), while 183 cases (80.62%) involved mixed infections. Similarly, mixed infections were predominant in children with non-severe pneumonia. These findings highlight the importance of employing comprehensive diagnostic methods capable of detecting a broad spectrum of pathogens in respiratory tract infections to minimize the risk of undiagnosed infections. This study identified the six most commonly detected pathogens in children with severe pneumonia: Human Respiratory Syncytial Virus, Cytomegalovirus, *Mycoplasma pneumoniae*, Rhinovirus, *Acinetobacter baumannii*, and Human Bocavirus. Severe pneumonia remains a significant threat to the health and survival of children (Wei et al., 2023; Cao et al., 2024). Additionally, severe pneumonia also imposes a substantial economic burden on families and society (Sultana et al., 2021). Timely vaccination against prevalent local pathogens, particularly those causing severe infections, can play a critical role in prevention (Meyerowitz et al., 2024; Rademacher et al., 2024). A comparison of pathogen distribution between severe and non-severe pneumonia groups revealed a higher detection frequency of Cytomegalovirus and Human Bocavirus in the severe pneumonia group. However, no significant differences were observed in the number of pathogens or infection patterns between the two groups.

Severe pneumonia can lead to respiratory issues like respiratory failure and lung abscess, as well as other complications such as myocardial damage and multi-organ failure (Severe Covid et al., 2020; Tang et al., 2023; de Miguel-Perez et al., 2024). These complications worsen the disease and significantly impact the patient’s prognosis. Studies have shown that the risk of complications in patients with severe pneumonia is closely related to the type of pathogen, the patient’s age, and pre-existing health conditions (Ni et al., 2022). For instance, patients with bacterial pneumonia frequently develop sepsis, resulting in a significantly higher mortality rate compared to those without sepsis (Liu and Li, 2022). In addition, complications like pneumothorax and respiratory failure may necessitate mechanical ventilation. This, in turn, increases the complexity and cost of treatment (Iqbal et al., 2023). This study revealed that 96.04% of children with severe pneumonia experienced respiratory complications, a rate significantly higher than the 55.95% observed in those with non-severe cases. The most common complications were respiratory failure (81.94%, 186 cases), pulmonary consolidation (132 cases), and hypoxemia (91 cases). Furthermore, 59.47% of children with severe pneumonia developed complications in other systems, compared to 29.52% in the non-severe group. Common complications included electrolyte disturbances (62 cases), gastrointestinal dysfunction (40 cases), anemia (35 cases), liver function damage (30 cases), and myocardial damage (23 cases). Therefore, prompt prevention, recognition, and management of these complications, particularly severe conditions such as respiratory failure, pulmonary consolidation, liver function damage, and myocardial damage, are essential to improving outcomes in children with severe pneumonia.

Prompt diagnosis of infectious pathogens is essential for effective disease management, improving patient outcomes, and preventing the spread of infection. Commonly used diagnostic methods, such as immunological testing and traditional PCR technology, have notable limitations, including low sensitivity, poor specificity, and the inability to detect multiple pathogens simultaneously (Tan et al., 2025). tNGS is a novel genetic sequencing technology designed to diagnose infectious diseases quickly and accurately by selectively capturing and amplifying the nucleic acids of specific pathogens. Studies indicate that tNGS can identify multiple pathogens with greater sensitivity and specificity. It is particularly effective in detecting complex infections and monitoring antibiotic resistance (Deng et al., 2023; Yin et al., 2024). This study compared the concordance of tNGS with traditional PCR and immunological detection methods. For detecting *Mycoplasma pneumoniae*, the concordance rates were as follows: tNGS with immunological methods was 60.48%, tNGS with PCR methods was 70.73%, and the combined use of both methods resulted in 81.71%. For the detection of RSV, the concordance rates were as follows: tNGS with immunological methods was 4.67%, tNGS with PCR methods was 88.06%, and the combined use of both methods also yielded 88.06%. The low detection rate of RSV-IgM antibodies can be attributed to several factors: (1) the immature immune systems of infants and young children, which reduce their capacity to produce antibodies and may result in false-negative test results; (2) the required window period for IgM antibody production, during which antibodies may be undetectable in the early stages of infection, leading to a low positive rate; (3) the sensitivity limitations of detection methods; (4) potential viral mutations; and (5) the use of diagnostic reagents that may not be optimally suited to the local population.

This study has several limitations. First, as a single-center study with a limited sample size, our findings may constrain the generalizability of the results, and subsequent validation in broader populations is necessary. Second, the enrollment period covered only five months of the year, failing to reflect full seasonal variations, which may affect the comprehensive assessment of the observed pathogen spectrum. Third, the targeted sequencing approach employed primarily focuses on known common respiratory pathogens and may therefore fail to detect certain rare or non-panel-included microorganisms.

In summary, children with severe pneumonia tend to be younger than those with non-severe pneumonia. Significant differences were observed between the two groups in major pathogen distribution, complications, clinical manifestations, and prognosis. However, no significant differences were found in the number of pathogens or infection patterns. These findings provide valuable insights for guiding vaccination strategies and have important implications for the clinical diagnosis, treatment, and management of severe pneumonia.

## Data Availability

The original contributions presented in the study are included in the article/**Supplementary Material**. Further inquiries can be directed to the corresponding authors.
