# Supplementary material for: Comprehensive insights into pathogen distribution, clinical features, and outcomes in pediatric severe pneumonia
Source: Front Cell Infect Microbiol. 2026 Jan 7;15:1681950. doi: 10.3389/fcimb.2025.1681950 (PMC12819815; doi:10.3389/fcimb.2025.1681950)
Supplement: Supplementary file 1 [file Table1.doc]

**Supplementary Table 1**. 198 respiratory pathogens (including 80 bacteria, 79 viruses, 32 fungi, and 7 other pathogens) detected by tNGS.

| **Microbial types** | **Pathogens** |
| --- | --- |
| **Viruses（79）** | BK polyomavirus (human polyomavirus type 1), human adenovirus type 11, human respiratory syncytial virus type A, human adenovirus type 1, human adenovirus type 14, human respiratory syncytial virus type B, human adenovirus type 2, human adenovirus type 34, human rhinovirus type 1 (human parainfluenza virus type 1), human adenovirus type 21, human adenovirus type 35, human rhinovirus type 3 (human parainfluenza virus type 3), human adenovirus type 5, human herpesvirus 6A, influenza A virus, human adenovirus type 55, human herpesvirus 6B, influenza A virus H1N1, human adenovirus type 57, JC polyomavirus (human polyomavirus type 2), influenza A virus H3N2, human adenovirus type 6, WU polyomavirus (human polyomavirus type 4), influenza A virus H5N1, human adenovirus type 7, Coxsackievirus A5, influenza A virus H7N9, human adenovirus type 3, Coxsackievirus A6, influenza B virus, human adenovirus type 4, Coxsackievirus B3, Coxsackievirus A10, human herpesvirus type 1 (HSV1), Echovirus E18, Coxsackievirus A16, human herpesvirus type 2 (HSV2), Echovirus E30, Coxsackievirus A2, human herpesvirus type 3 (VZV), enterovirus, influenza C virus, human herpesvirus type 5 (CMV), enterovirus group A, influenza A virus H1N1 (2009), human herpesvirus type 6, enterovirus A71, influenza B virus Victoria lineage, human herpesvirus type 7, enterovirus group B, influenza B virus Yamagata lineage, human bocavirus type 1, enterovirus group C, measles virus, human bocavirus type 2, enterovirus group D, mumps virus, human bocavirus type 3, enterovirus D68, rhinovirus, human bocavirus type 4, human coronavirus 229E, rhinovirus type A, human herpesvirus type 4 (EBV), human coronavirus HKU1, rhinovirus type B, human adenovirus, human coronavirus NL63, rhinovirus type C, human adenovirus group B, human coronavirus OC43, rubella virus, human adenovirus group C, human metapneumovirus, SARS-CoV-2 , human adenovirus group D, human parainfluenza virus type 2 (human mumps virus type 2), human parvovirus B19, human parainfluenza virus type 4 (human mumps virus type 4). |
| **Bacterium（80）** | Corynebacterium diphtheriae, Nocardia saintgeorgensis (Nocardia gelsenkirchenensis), Klebsiella aerogenes, Mycobacterium asiaticum, Nocardia farcinica, Klebsiella oxytoca, Mycobacterium avium, Nocardia nova, Klebsiella pneumoniae, Mycobacterium avium complex (MAC), Nocardia otitidiscaviarum, Klebsiella variicola, Mycobacterium occultum, Nocardia terpeneae, Legionella spp., Mycobacterium gordonae, Micromonospora parva, Legionella bozemanii, Mycobacterium intracellulare, Rhodococcus equi, Legionella pneumophila, Mycobacterium kansasii, Staphylococcus aureus, Legionella longbeachae, Mycobacterium malmoense, Streptococcus agalactiae, Legionella micdadei, nontuberculous mycobacteria (NTM), Streptococcus anginosus group, Moraxella catarrhalis, Mycobacterium scrofulaceum, Streptococcus intermedius, Acinetobacter baumannii, Mycobacterium schroeteri, Streptococcus pneumoniae, Acinetobacter johnsonii, Mycobacterium simiae, Streptococcus pyogenes, Acinetobacter ursingii, Mycobacterium szulgai, Tropheryma whipplei, Bacteroides fragilis, Mycobacterium tuberculosis complex, Peptostreptococcus anaerobius, Homburgeria, Mycobacterium ranarum, Burkholderia cepacia complex, Bordetella parapertussis, Mycobacterium abscessus, Burkholderia mallei, Bordetella pertussis, Mycobacterium chelonae-abscessus complex, Burkholderia pseudomallei, Brucella spp., Mycobacterium chelonae, Burkholderia contaminans, Burkholderia cepacia nova, Mycobacterium fortuitum, Burkholderia multivorans, Burkholderia cepacia, Mycobacterium smegmatis, Elizabethkingia anophelis, Neisseria meningitidis, Nocardia spp., Elizabethkingia meningoseptica, Pasteurella multocida, Nocardia abscessus, Enterobacter cloacae complex, Proteus mirabilis, Nocardia africana, Escherichia coli, Pseudomonas aeruginosa, Nocardia asteroides, Fusobacterium necrophorum, Serratia marcescens, Nocardia brasiliensis, Fusobacterium nucleatum, Stenotrophomonas maltophilia, Nocardia cavae, Haemophilus influenzae. |
| **Fungi (32)** | Candida glabrata, Fusarium spp., Rhizomucor pusillus, Aspergillus flavus complex, Histoplasma capsulatum, Rhizopus spp., Aspergillus fumigatus, Mucorales spp., Rhizopus delemar, Aspergillus niger complex, Mucor plumbeus, Rhizopus microsporus, Aspergillus terreus complex, Mucor ramosissimus, Rhizopus oryzae, Candida albicans, Candida guilliermondii (Pichia guilliermondii), Cephalosporium spp., Candida pseudoglabrata, Mucor irregularis, Cephalosporium acremonium, Candida parapsilosis, Mucor racemosus, Cephalosporium boydii, Candida tropicalis, Pichia kudriavzevii (Candida krusei), Talaromyces marneffei, Cryptococcus gattii, Pneumocystis jirovecii, Trichosporon asahii, Cryptococcus neoformans, Mucor rhizopus. |
| **Others (7)** | Pneumonia chlamydia, Mycoplasma pneumoniae, Coxiella burnetii, Chlamydia psittaci, Ureaplasma parvum, Chlamydia trachomatis, Ureaplasma urealyticum. |
